# An integrative review of the impact of allied health student placements on current staff’s knowledge and procedural skills in acute and primary care settings

**DOI:** 10.1186/s12909-024-05632-7

**Published:** 2024-06-12

**Authors:** Mohammad Hamiduzzaman, Sarah Miles, Sarah Crook, Lewis Grove, Jennie Hewitt, Frances Barraclough, Peter Hawkins, Erika Campbell, Nicola Buster, Kate Thomson, Christopher Williams, Vicki Flood

**Affiliations:** 1 University Centre for Rural Health (UCRH), School of Health Sciences, Faculty of Medicine & Health, The University of Sydney, Lismore, NSW Australia; 2 School of Health Sciences, The University of Sydney, Sydney, Australia

**Keywords:** Clinical training placements, Aged care staff, Knowledge, Procedural skills, Collaborative learning, Rural health

## Abstract

**Background:**

Staff shortages limit access to health services. The bidirectional benefits of allied health clinical placements are understood in the domains of student learning, health service delivery, and future workforce development. Still, the benefits to current workforce outcomes remain unknown. This review provides insights into the effects of allied health student placements in acute and primary care settings, particularly on healthcare staff's knowledge and procedural skills.

**Methods:**

This search was based on the integrative review process established by Whittemore and Knafl in 2005. In October 2023, the first author (MH) searched five major electronic databases: Medline-EBSCO, PubMed, CINAHL, Embase, and Scopus. The CLUSTER model was used to track additional references. The first three authors (MH, SM, and SC) were involved in screening, quality appraisal, and synthesis of the studies. Data were thematically synthesised and analysed.

**Results:**

MeSH headings and keywords were used in key search areas: health education, health professional training, clinical placements, and allied health professions. The systematic search yielded 12 papers on allied health student placements across various healthcare settings in rural and metropolitan areas, with no high-quality methodologies measuring student placements' impact on staff knowledge and skills. Four main themes were identified from the analysis: meaningful student integration in service delivery, targeted educational support to healthcare staff, development of staff procedural skills and confidence, and the mechanisms of why student placements work in this aspect.

**Conclusions:**

This review suggests that offering allied health student placement could be a promising approach to supporting rural healthcare staff in performing patient assessments and treatments proficiently and collaboratively. However, this requires further investigation to confirm.

## Introduction

Healthcare staff shortages limit access to health services [1]. Four key areas for immediate attention in the Australian health context are food and nutrition, dementia care, the use of restrictive practices, and palliative care [2]. Allied health professionals have an important role to play in each of these areas. However, there is a critical shortage of allied health professionals and a higher turnover rate among allied health workers across Australia [2, 3]. This shortage becomes more pronounced as the number of healthcare staff decreases with increasing remoteness [3]. Health service disparities persist between rural and metropolitan areas in Australia, with a gap in life expectancies (78 years compared to 82.5 years), a prevalence of chronic disease (21% vs 18% per 100,000 population), and potentially avoidable death rates (775.9 deaths vs 587.9 deaths per 100,000 population) [1, 4]. Current funding and employment models have led to issues with recruitment and retention of allied health professionals and a shortage of staff [5, 6]. For example, in 2018–19, only 29% of Australians used allied health services [7]. An additional challenge to upskilling healthcare staff is a lack of professional development opportunities [8, 9]. Student placements have been identified as a potential approach for health workforce capacity building and support of health services delivery, especially in rural areas [9–11].

Various clinical training placement models exist to facilitate learning opportunities for medicine, nursing, and allied health students by integrating them into health service delivery for patients [12]. These placement models include practice-based learning [13], experiential learning [14], service-learning [15], work-integrated learning [16], and integrated clinical placements [17]. Clinical placements benefit students, educational institutions, and healthcare organisations in different ways, including personal growth and professional experience for students, academic rigour and service to the community for universities, and a workforce fit to practice in healthcare organisations. Evidence shows that clinical placements of students with exposure to acute and primary healthcare contexts are associated with better impacts in terms of students’ intellectual transformation [18–20], workforce capacity building [21–23], and patient health outcomes [24, 25]. There remains a notable gap in research on allied health student placements that builds staff capability.

Educational and training resources designed for clinical supervision of allied health students during their placements can also serve as professional learning opportunities for healthcare staff. Professional development is imperative for healthcare staff to stay up to date with knowledge and technical skills and create innovative treatment planning. Complex and infrequently used clinical skills often deteriorate among health professionals, as confirmed in a systematic review by Main and Anderson [25] in Australia [26]. The National Health Workforce Strategy advocates for continuing professional education and training for health professionals so that professionals “maintain, improve, and broaden their knowledge, expertise, and competence, and develop the personal and professional qualities throughout their professional lives” [27]. Healthcare professionals have reported that ongoing education and training opportunities have improved their knowledge and procedural skills in client (e.g., patients, residents in aged care homes) care [28]. Since the COVID-19 pandemic, access to online professional development modules and training has improved [29]. However, a lingering question persists: can the co-creation of training programs and educational modules effectively contribute to the knowledge and skills development of both allied health students and healthcare staff?

A compelling association exists between student placements, health workforce capacity and capability building [30]. As noted earlier, student placements contribute to workforce recruitment and retention in rural and metropolitan areas by immersing them in health and social care settings. Throughout placements, students benefit from access to tutorials and clinical supervision [12, 15, 16]. Additionally, students and healthcare staff from different disciplines work collaboratively in a team during placements [31]. Pedagogical frameworks, including social learning theory [32], social constructivism [33], interprofessional learning [34], and community of practice [35] suggest that individuals working together learn with and from one another. The Royal Commission into Aged Care Quality and Safety in 2021 recommends strengthening allied health services [2], particularly in rural areas; therefore, a review of existing literature is important to inform how and why the placements work to enhance the capability of healthcare staff in service delivery.

### Aims of the study

This review aims to synthesise the effects of allied health student placements on healthcare staff's knowledge and procedural skills in acute and primary care settings.

Two main questions guided this review:

Q1: How do the studies describe the integration of allied health students in services design and delivery in acute and primary care settings?

Q2: How do these studies describe the effectiveness of allied health student placements for current healthcare staff’s knowledge and procedural skills in acute and primary care settings?

## Methods

This review adhered to the five steps of an integrative review process as its foundation, established by Whittemore and Knafl in 2005 [36]. These steps included problem identification, literature search, data evaluation, data analysis, and presentation. We systematically searched the literature and employed the Mixed Method Appraisal Tool (MMAT) to assess the quality and rigour of the selected papers [37]. The extracted data were then analysed and presented thematically.

### Search strategy

The systematic search for published documents was conducted following the PRISMA guidelines [38]. In October 2023, the first author (MH) searched five electronic databases: Medline-EBSCO, PubMed, Embase, CINAHL, and SCOPUS. A combination of MeSH headings and relevant concepts was used in crucial search areas: health education, health professional training, clinical placements, and allied health professions (the full search strategy is available in Table 1). The CLUSTER model was also employed to track sibling studies and citations for supplementary references.

**Table 1:** Search Strategy to identify papers for this review (CINHAL)

Step	Search term	Results
1	Health educat*	159,483
2	Health professional training*	112
3	S1 OR S2	159,483
4	(“model* n2 (placement* OR clinical education OR clinical learning OR service learning placement OR clinical placement* OR clinical practice or “interprofessional learning”)	2,089
5	(MH "Simulation training")	1,615
6	S4 OR S5	3,696
7	(Allied health profession* n2 (physiotherapy* OR occupational therapy* OR dietician* OR speech pathology* OR exercise physio* OR social work* OR optometrist* OR podiatrist* OR psychologist* OR osteopath* OR))	48,744
8	S3 AND S6 AND S7	1,264
9	Language: English	1,091
10	Date range 2001 onwards	717

*(Truncation) - By placing an asterisk at the end of a word root, the search includes any word that starts with that root

### Inclusion and exclusion criteria

The clinical placements are typically designed to immerse health students in real-life experience in acute and primary care settings with the aim of future workforce recruitment. Given the specific focus of this review on the impact of allied health student placements on the knowledge and procedural skills of existing healthcare staff, medical and nursing professions were not included in the search. The search was also limited to certain allied health disciplines based on the discussion with allied health clinicians and health service providers, such as physiotherapy, occupational therapy, dietetics, speech pathology, exercise physiology, social work, optometry, podiatry, psychology, and osteopathy. The inclusion criteria were articles and reports published in English, publication year 2001 to the present, descriptions of actual allied health student placements, and the placements aimed at enhancing the capacity and capabilities of current healthcare staff. Aligning with this review’s objectives and considering the scarcity of studies conducted in rural locations, the search was not restricted solely to rural placements. While the primary outcomes of allied health student placements predominantly centred on student learning, patient health and wellbeing, and workforce recruitment and retention, the studies that explored these aspects as their primary focus were not excluded when they identified the placements’ contribution to healthcare staff. Two reviewers, MH and HG independently screened the records retrieved by title, abstract, and full text. Discrepancies were discussed with a third reviewer, SM.

### Quality appraisal

The MMAT criteria were used to assess the quality of studies, using a scale that spanned from 0, indicating no criteria met, to 5, indicating all criteria met, as detailed by Hong et al. in 2018. [37] To evaluate the studies, two reviewers, MH and HG, conducted separate assessments, allocating scores out of 5 (0—Unclear/No and 1: Yes). Through a consensus-driven process, it was determined that the papers included in this review exhibited a quality level that ranged from moderate (with a score of 3) to high (with a score of 5), as indicated in Table 2.

**Table 2:** *S*tudies evaluating the impact of SLP for older patients with preventable chronic conditions in Australia

Author (year)	Study design	Placement Settings	Study participants	Integration of allied health students in service delivery			Placement outcomes		MMAT scores
				**Placement focus and participants**	**Types of placements**	**Level of integration**	**Impact**	**Limitations**	
Buchanan, Jenkins, and Scott [39]	Report Interviews	Australia Health settings	Key informants *N* = 26	Explore the context, structures, operations and characteristics of placements in the health and clinical sciences	Dentistry, exercise physiology, medicine, nursing and midwifery, nutrition and dietetics, occupational therapy, pharmacy, physiotherapy, psychology, radiation science, rehabilitation counselling, social work, and speech pathology	General administrative tasks, assisting with administering and refining quality assurance systems, creating resources like patient education materials and brochures, management of work waiting list/patient scheduling, and in advanced cases, assistance with review and evaluation of work processes and flowsDirect engagement in clinical service delivery, especially in rural areas	Functional improvements in service delivery Numerous activities during the placements each year supported staff (across allied health, medicine and nursing disciplines) in core areas of learning Implementing 'Grand Rounds' in several hospitals provided learning and networking opportunities Service based learning, professional development and training in education and supervisory roles for LHD staff are not discrete services provided in a fee for service basis – rather they are an ensemble of activities that benefit all parties concerned Prompts staff reflection and clinical reasoning through own practice Staff went back to basics Reorientation with the assumptions and theory behind the interventions	Not reported	3
Johnston et al. [40]	Cross-sectional study Survey	Australia Residential aged care homes (Regional and rural)	Facility managers (*N* = 40) Physiotherapists (*N* = 25) Dieticians (*N* = 1)	Allied health management in residential aged care homes Physiotherapy and Dietetic students	Student clinical placement Students from physiotherapy dietetics	Developing treatment plans Engaged in delivering services to the residents	There was no significant difference between managers and allied health professionals in reporting whether they thought there were advantages (*p* > 0.05), or barriers (*p* > 0.05) to placements in aged care More allied health professionals than managers indicated there were disadvantages; however, this was not statistically significant (*p* > 0.05) Majority of allied health professionals indicated that student placements would have a positive influence on preparedness to work in this field Managers suggested that student placements within their facilities could provide an opportunity for other staff to improve their knowledge and skills	Training for staff to supervising students	4
Kemp et al. [41]	Interpretivism and social exchange theory Qualitative study Interviews	Australia Community-based organisations (local government areas, community health service, non-government health organisation, primary care partnerships, non-government non-health organisation)	Clinical educators *N* = 17	Public health nutrition (food insecurity, health food access and childhood obesity) Undergraduate and postgraduate student dietitians	Work-integrated learning 7 weeks duration	Assess priorities Not engaged in delivery of services Plan and evaluate interventions	Demonstrated capacity of advancing projects, tasks, and action plans that the existing staff lacked the staffing and time to accomplish The staff work scope was extended and broadened the directions for new projects because of increasing staff capacity and reduced workload Accelerate the time frame of current service delivery Creation of new data and new knowledge from local evidence that didn’t exist	Student capabilities varied because of a lack of professionalism and communication skills	4
Lauckner et al. [42]	Pre- and post-test surveys Focus groups Interviews	Canada Nursing homes	Students (*N* = 24) Staff (*N* = 30) Family members (*N* = 5) Resident (*N* = 1)	Training in geriatric care and long-term care Undergraduate students from Pharmacy, Social Work, Therapeutic Recreation and Dietetics programs, and graduate students from Speech-Language Pathology and Occupational Therapy	Interprofessional team placement Interprofessional education 4–6 weeks of duration	Students participated in a shadowing experience with a front-line care worker and a long-term resident Implementation of staff orientations For 1 to 2 h the student shadowed the care worker and then they spent 1 to 2 h with a resident the care worker deliver services	Collaborative practice was instrumental in less hierarchical culture within the care setting A cultural shift towards more collaboration among staff	Staff were hesitant about the hierarchical setting that could impact decision-making and teamwork	5
Longman, Barraclough and Swain [43]	Survey	Australia Rural community-based organisations (preschool, school and aged care facility)	Allied health students *N* = 163	Cultural and social equity education, providing continuous service throughout the year, and quality improvement initiatives in placement sites Undergraduate and postgraduate, occupational therapy, physiotherapy and speech pathology students	Service-learning placement Interprofessional education 4–10 weeks duration	Students worked autonomously, used their clinical reasoning and problem-solving skills Need assessment Direct engagement in delivery of services, exercise programs, sensory garden, craft and cooking Plan and evaluate interventions Engaged in staff education on improving management and quality of life of residents	An increased the quantity of interventions Increased staff understanding of the roles of allied health disciplines and the positive impacts they could have on resident quality of life	Working autonomously was a challenge for some students	4
MacBean et al. [44]	Delphi study Surveys Focus groups Professional forums	Australia Clinical Skills Centres or Patient Training Centres	Professionals (clinical educators, recent graduates, and management) Students	Increase student placement capacity Undergraduate and postgraduate students from speech pathology	Simulated learning environment Interprofessional education Not reported	Communication Need assessment Clinical reasoning History taking Planning interventions, and evaluations	The positive impact of students within a workplace was noted, including increased workplace productivity and expanded service delivery Additional exposure to specialized areas of speech-language pathology practice Clinical placement learning models have potential to improve the ongoing professional development of current clinical staff	More resources required included: adequate staffing, development, and recur-rent funding, appropriate teaching space, and appropriate materials	4
Mu et al. [45]	Pre- and post-test research Survey Qualitative data collection (preservation assessment, on-site summary, post observation assessment, reflection journal and post experience debriefing	USA Rural Health services	Students *N* = 111	Develop a new and innovative model for interprofessional student training Students from physiotherapy, occupational therapy and pharmacy	Interprofessional rural training	Level 1—Observation role Level 2 – direct engagement in delivery of services Level 3 – management	Inter-professional team enables health staff to set one’s ego aside for the patients’ good Benefits of interprofessional work included enhancing communication among health staff, staff to share information, learning from and helping each other in providing health care services, and avoiding overlapping or contradictory services Participants voiced that they were amazed to see physical therapists and occupational therapists work together during therapy sessions and found that they are capable of switching roles throughout the session	Students voluntarily supported the residents Maturity and learning experience of students	5
Nguyen et al. [46]	Benefit–cost analysis	Australia Cities, regional and rural Residential aged care homes	Cos: initial investment and the operating cost Benefits to reference group	Integrating students to provide optimal care for older adults Graduate students from Dietetics, Occupational Therapy, Physiotherapy, Pharmacy, Medicine, and Nursing	Interprofessional clinical placements	Engaged in delivering care, including clinical assessments, case studies, ward rounds and training sessions	Staff were benefited from reduced absenteeism and turnover Staff were engaged in teaching, facilitating, and managing students	Never present an attractive investment from a market perspective	3
Nisbet et al. [47]	Pragmatism Case study design Focus groups and interviews Patient experience surveys Secondary administrative records	Australia Acute care settings	Student focus groups (*N* = 21) Educator interviews (*N* = 19) Manager and clinician focus groups (*N* = 6) Patient surveys (*N* = 91)	Integrating students into service delivery Undergraduate and postgraduate, physiotherapy and occupational therapy students	Service-focused placement model Placement blocks: 22 5–8 weeks duration	Delivery of services in inpatients, outpatients, and a range of wards	Health staff perceived efficiencies in delivering services in relation to patient flow, through the health system, flexibility in care provision, and re-distribution of staff resources Students were considered to have contributed to earlier patient readiness for discharge further supporting improvements in patient flow Staff had the flexibility to see patients at different stages in the patient journey (e.g. inpatient then outpatient service) and via different modalities (e.g. group versus individual care) Efficiencies were also gained through continuity of care, timeliness of that care and its seamless delivery Increased the capacity of health staff to see more patients	There was a concern that, without students, changes were unlikely to be sustained Early review of patients	5
Reid and Barbaro [48]	Case study Feedback	Australia Rural health service	Students and staff Participant number: Not reported	Integrating students into primary care services	Student placement Interprofessional education	Assess priorities Explore health literacy among rural young people Contributed to strategic planning Not engaged in operational activities	Students collected valuable data that staff can continue to use for strategic planning and prioritising OT staff enjoyed the student placement and sharing the placement worked well	Many rural allied health positions are part-time and often staff can be the sole clinician for their discipline. This leads to lack of time and resourcing to support students and adds burden to the clinician’s already busy role	4
Seaman et al. [49]	Mixed methods evaluation report Surveys Focus groups and interviews	Australia Cities, regional and rural Residential aged care homes	Students (*N* = 453)	Integrating students to provide optimal care for older adults Graduate students from Dietetics, Occupational Therapy, Physiotherapy, Pharmacy, Medicine, and Nursing	Interprofessional clinical placements	Engaged in delivering care, including clinical assessments, case studies, ward rounds and training sessions	Ninety-two percent of staff believed the program was beneficial to themselves and their colleagues Over half of staff believed their knowledge and skills had improved as a result of working with students at their facility Staff were appreciative of the knowledge that students undertaking interprofessional placement provided them with both formally and informally, ultimately improving the care of residents by increasing capacity of existing staff The knowledge and capacity of staff was increased during the interprofessional education program due to the extra training and resources provided and the staffs’ interactions with students Students provided staff with current and new information they otherwise would not have accessed Staff attended training sessions with students that were delivered by students or professionals on topics relevant to aged care such as ‘mealtime positioning’, ‘positioning post stroke’ and ‘medication in palliative care’. Staff providing feedback on training often ‘agreed’ or ‘slightly agreed’ they had increased their professional knowledge (79%) Students also challenged staff to be aware of evidence-based practice Some staff noted that following training sessions they changed their practice to be in accordance with their new knowledge such as ‘check position of residents more’ following seating and positioning training Staff also expressed their increased awareness of topics such as medication and commented they would be more mindful of these in future practice	Concern regarding sustainability	5
Seaman et al. [50]	Mixed methods evaluation Surveys Focus groups and interviews	Australia Cities, regional and rural Residential aged care home	Staff (*N* = 45) Residents (*N* = 15) Family and friends (*N* = 35)	Integrating students to provide optimal care for older adults Graduate students from Dietetics, Occupational Therapy, Physiotherapy, Pharmacy, Medicine, and Nursing	Interprofessional clinical placements	Engaged in delivering care, including clinical assessments, case studies, ward rounds and training sessions	The placements had provided assistance to the staff in care delivery Highly beneficial to staff in terms of learning and affirmation of abilities Interprofessional placement was found very good for developing team skills	Lack of sustainability caused emotional distress among the residents Direct care staff wanted students to consult them more often when appropriate and relevant Longer placements were preferred	4

### Data extraction and analysis

Three reviewers, MH, SM, and SC, read the papers meeting the inclusion criteria multiple times to extract data. The extracted data were recorded separately by these three reviewers into Excel spreadsheets, with any discrepancies carefully cross-checked (Table 2). The extracted data included the study characteristics (author, year, country of origin, study design, study participants); characteristics of allied health student placements (placement setting, focus, participants, type of placement, the level of student involvement in service delivery); outcome data for existing healthcare staff’s knowledge and skills, as well as the limitations of these placements. Given that the selected studies were heterogeneous in methodologies, a thematic data synthesis was deemed the most appropriate approach [45]. The categories and sub-themes were independently identified by the reviewers, MH, SM, and SC, and were subsequently deliberated upon during review team meetings to determine the final themes and validate interpretations.

## Results

**Fig. 1:**
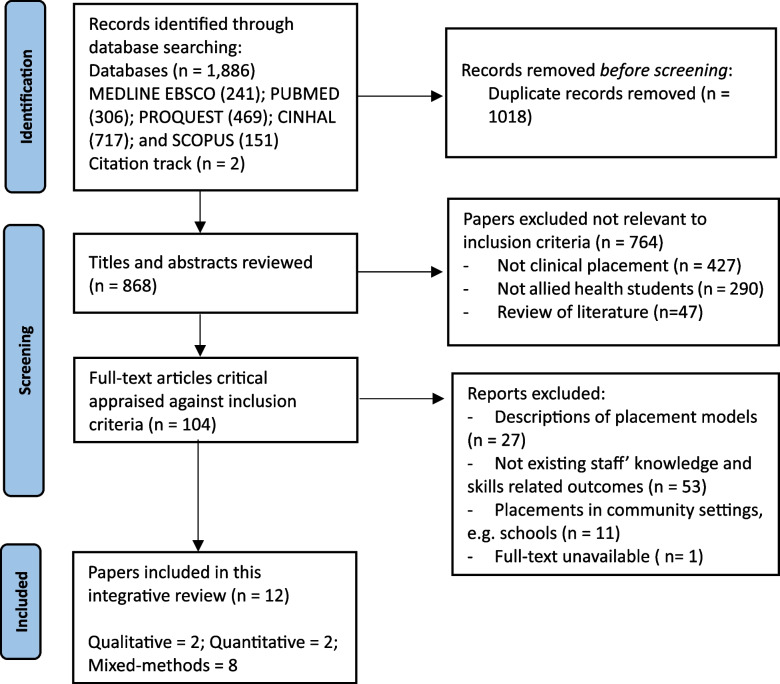
PRISMA 2020 flow diagram of systematic search and selection process

Figure 1 illustrates the selection process of the studies reviewed. Twelve papers that met the inclusion criteria represented the highest number over the past decade. Among these, eight studies used mixed methods for evaluating the placements, while two were qualitative and two were quantitative methodologies. The selected placements were mainly in Australia (10), with all papers originating from high-income countries, including the USA (1) and Canada (1). The healthcare settings were diverse across the placements; half were in residential aged care homes, while the rest were in hospitals, community health services, clinical skills centres, patient training centres, and non-government health organisations. The study participants included students, patients/residents, healthcare staff, health service managers, clinical educators, and relevant key stakeholders like family members and community organisations. Rural placement was reported in the majority of studies (7), but no studies compared the effects of different locations.

All twelve studies focused on either allied health student learning outcomes or service delivery across a range of settings by placing students. Most placement programs narrowly focused on the professional development of existing healthcare staff, while exclusive focus on this aspect was identified in four placement programs facilitated in hospitals, residential aged care homes, and community health services [39, 47, 49, 50]. Undergraduate and postgraduate students from different allied health disciplines participated in the placements, including physiotherapy, occupational therapy, nutrition and dietetics, social work, and speech pathology. Some studies featured the collaboration between medicine, nursing, and allied health students [40, 46, 47, 50]. Various types of placements were discussed, such as clinical placement [41, 48]; work-integrated learning [42]; interprofessional team placement [40, 43, 49]; service-learning placement [39, 44, 47, 50]; and simulated learning [51]. Interprofessional education was reported in most of the studies (8), and four studies provided information on the duration of placements, which ranged from four to ten weeks; in addition to detailing the types and focuses of the placements, the synthesis of outcome data revealed four key themes.

### Meaningful student integration in service delivery

The integration of allied health students in health service delivery for patients was identified as a powerful and essential part of all placement programs. Student involvement in health service delivery was described by their engagement in a wide range of activities, from administration tasks and priority assessments to developing and implementing treatment plans and evaluating interventions. Eight studies reported direct engagement of students in developing treatment plans and designing and delivering services. Examples included person centred exercise programs, developing a sensory garden, implementing craft and cooking sessions for residents with dementia and training and upskilling care staff [39, 40, 44, 46–50]. In contrast, four placement programs were restricted to organisations’ priority assessments [41, 42, 51]; shadowing a care worker and spending time with residents [43]; and planning and evaluation of interventions [41, 42, 51]. Student involvement in delivering direct health services to patients was identified in both urban and rural healthcare settings.

The extent of students’ involvement in delivering health services to patients was somewhat related to the degree to which the placement supported the capacity and capability building of existing healthcare staff. Integrating students in administrative tasks, priority assessments, and evaluation of the treatments contributed to staffing management and timely task completion, as well as a cultural shift towards collaboration among the staff [41–43, 51]. Direct engagement of students in treatment plans and patient/resident care management was highly beneficial to a healthcare staff’s reflection and clinical reasoning [39, 40, 44, 46–48, 50]. Of note, none of the studies measured the causal relationships between the level of student integration in service delivery and the professional development of healthcare staff.

### Targeted education support to healthcare staff

All studies reported that the placements led to an increase in knowledge, or had the potential to do so, for both students and healthcare staff. During these placements, various learning activities were offered to students, which, in turn, enhanced the knowledge of healthcare staff. For instance, learning activities like Grand Rounds and interprofessional education were implemented [44, 46, 47, 50, 51]. Key areas of learning for healthcare staff were identified in one evaluation study of interprofessional team placement in residential aged care homes [50], including mealtime positioning, post-stroke positioning, and medication management in palliative care. Additionally, one qualitative study described how the placements allowed healthcare staff to reorient themselves with the theories and methods behind the treatments [46]. Attending education and training sessions also helped the rural healthcare staff become familiar with the roles and responsibilities of other health disciplines [44].

Three studies reported that students generated new data and knowledge based on local evidence during their placements [41, 42, 50]. Two of the studies included rural placement of students [41, 50], but all the studies confirmed that the students provided healthcare staff with current and innovative knowledge. This new knowledge supported the staff in strategic planning and prioritising patient assessments and treatments.

### Development of staff procedural skills and confidence

Eight studies highlighted that allied health student placements were useful in developing procedural skills among healthcare staff. In four of these placements, student training sessions enhanced the healthcare staff’s efficiency in service delivery by reorienting them with the standards and procedures of the treatments [39, 46, 49, 50]. Healthcare skills development various skills, including critical reflection, clinical reasoning, patient flow management, timely assessment and treatment of patients, continuity of care, clinical communication, patient safety, and evidence-based practice. The Delphi study conducted by MacBean et al. [43] in inpatient training centres in Australia provided insights into how the placements broaden the healthcare staff’s scope of practice in speech pathology, which was further complemented by the qualitative study of Kemp et al. [41] in Australian community health services. [42, 51] Healthcare staff also gained confidence in performing clinical tasks during the student placements, with their abilities being questioned and affirmed [46, 47, 50]. Interprofessional team placements were found to be effective in two studies for team skills development [49, 50]. Both rural and urban healthcare staff benefited equally from student placements in healthcare settings.

### Why do student placements work? Insights into the mechanisms

This review identified the mechanisms underlying how the allied health student placements supported the professional development of healthcare staff in seven studies. While a cross-sectional study indicated non-statistically significant disadvantages of student placements in regional and rural residential aged care homes [48], six studies, spanning various healthcare settings, reported functional improvements in health service delivery attributed to student placements [39, 40, 42, 46, 49, 50], regardless of the locations. These functional improvements in service delivery were because of additional training and resources, as well as active engagement in teaching, facilitating, and managing students within healthcare settings, which were identified as supportive for healthcare staff’s professional development [40, 46, 50]. Collaborative practice was found to be instrumental in reducing hierarchical culture among healthcare staff [43, 49]. Additionally, the placements contributed to early patient readiness for discharge, providing staff with flexibility in using client care modalities, and questions from students increased staff awareness of evidence-based practice [39, 50].

**Fig. 2:**
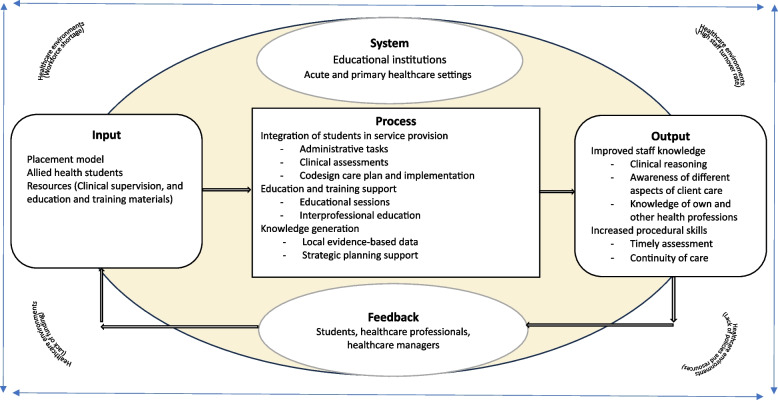
Integration of allied health students in healthcare settings and its impact within a system theory framework

In order to facilitate discussions, the findings of this review are positioned within a general system theory framework (Fig. 2), enabling the assessment of inputs, transformational processes, outputs, and the environment within acute and primary healthcare settings.

## Discussion

The role of allied health student placements in fostering professional development of healthcare staff is promising, with most of the studies in this review showing positive evidence. Service-based placements, with a meaningful integration of students in health service delivery, show the most potential. Service-based placements might work by offering Grand Rounds and interprofessional education sessions to healthcare staff in critical areas of client care, generating new knowledge that can form powerful local evidence, and enhancing healthcare staff's understanding of other health professionals and service providers that can promote the collaborative practice. Regardless of the locations, active engagement in supervising and educating students and increasing awareness of training sessions have proved to be beneficial for healthcare staff in developing their professional knowledge and skills in client care.

There is a strong evidence base for the integration of allied health students into various aspects of client care, but engagement has varied. Student involvement in service delivery can be particularly powerful as it primarily emphasises the improvement of patient accessibility and utilisation of health services that are otherwise not accessible to them, especially in rural communities [52, 53]. In the studies included in this integrative review, students played vital roles in the development of treatment plans, treatment of patients, and evaluation of interventions, and this integration was found to be beneficial to current health workforce capacity and capability building. Previous placement programs involving medical and nursing students corroborate the positive outcomes, citing the development of confidence and proficiency in both students and healthcare staff [54, 55]. These programs recognised the bi-directional benefits of clinical placements. Since 2021, the Rural Health Multidisciplinary Training (RHMT) in Aged Care Program has supported University Departments of Rural Health (UDRHs) in Australia to expand their capacity to facilitate health student placements in aged care settings. This review is timely to inform clinical educators by providing insights to design education sessions that meet the learning needs of students and staff.

Within the limited number of studies available for review**,**education sessions during student placements appear to be important for developing professional knowledge and skills of healthcare staff. This review strengthens the previous study findings in medicine and nursing placements in acute care settings, stating that Grand Rounds and interprofessional education opportunities increased healthcare staff and students’ awareness of different aspects of client care and expertise of their own and other professions [56–59]. These ongoing sessions cover various aspects of client care and are likely to equip staff with theories behind the treatments. Rural healthcare staff often have limited access to professional development opportunities, as well as supervision of students that has the potential to add a new perspective to the staff workloads [11, 59, 60]. Rural healthcare staff in community settings may also have limited time to engage with professional learning opportunities in their normal work routine, so embedding opportunities for ongoing education in the workplace through student placements may be beneficial. Opportunities must be explored in collaboration with healthcare and community partners to ensure professional development and training is co-designed and co-delivered to meet their staff’s unique needs. Creating ongoing learning opportunities for staff and engaging them in student supervision is vital to the success of placements.

In terms of creative learning, the student placements’ contribution to generating new and local evidence emerges with some supporting findings. Many studies explored how students are engaged in reciprocal learning relationships with peers and healthcare staff in the domains of clinical knowledge and procedural skills [58, 61]. Students bring new or different perspectives, up-to-date knowledge of evidence-based practice, do not have the workload expectations, and are not restricted by funding requirements. This allows students to bring a different perspective. Students often have more time to complete projects and create resources, and when co-designed with staff and patients, such resources can enhance both staff learning and patient outcomes. However, these bi-directional learning benefits receive less attention from educators and rural health service providers. It may be unclear what students could add to the knowledge and skills of staff who are already registered and experienced in delivering services. Evidence is limited on how to design education sessions for different learner groups.

The review suggests that active engagement of healthcare staff is often absent in student placements. While clinical educators currently take the responsibility for student supervision and management, a potential improvement could involve active engagement of healthcare staff in these aspects during placements, which may help address the two remaining questions. First, whether it is important to create collaborative learning environments before offering student-led education of staff. This could enhance understanding and knowledge of both staff and student roles, increasing collegiality and co-design of learning and knowledge. A second question is whether adding a co-supervision role for healthcare staff in the allied health student placements (by adapting the models of medicine and nursing placements in rural communities) is a viable option to enhance staff engagement. This role could upskill the current health workforce in rural areas, increasing the capacity to take student placements. This role may combine rural knowledge with an understanding of student models and seek to implement changes in practices developed from student placements.

### Limitations

Developing the search strategy was challenging because of the diversity in placements, disciplines, settings, and associated terminology. This resulted in a search that yielded only 12 eligible studies for review. Since allied health student placements in rural healthcare settings have expanded across high-income countries in recent years, there will likely be articles under review about unsuccessful placements that could have provided additional insights. Further rigorous investigations are required to strengthen the evidence surrounding student placements’ contribution to improving rural health staff knowledge and procedural skills in client care. These investigations could delve into the unique workforce outcomes associated with individual allied health disciplines and consider the different levels of study among students (undergraduate vs postgraduate).

## Conclusions

This review is the first synthesis of the impact of allied health student placements on the professional development of our current health workforce. To enhance staff knowledge and skills and address shortages, particularly in rural and remote communities, this review indicates the importance of student integration in the delivery of health services. A collaborative learning approach to increase the knowledge of students and staff and improve staff engagement in placements that promote interprofessional learning is key to the professional development of current staff in any healthcare setting. While there is little evidence of the generation of new knowledge by students during their placements, there is no indication that these placements disadvantage healthcare staff in relation to their professional development. Clinical educators may consider establishing co-supervision roles for rural healthcare staff to foster interactions between staff and students and to enhance positive learning experiences for both parties. Individually tailored and co-designed professional development opportunities could be important, for instance, to assist rural healthcare staff in reducing adverse events and ensuring adequate health services and the quality of integrated care.

## Data Availability

All data generated or analysed during this study are included in this article.
